# The dimensionality of phonological awareness among Brazilian Portuguese-speaking children: a longitudinal study

**DOI:** 10.1186/s41155-021-00192-x

**Published:** 2021-08-03

**Authors:** Cláudia Nascimento Guaraldo Justi, Flávia Guimarães Henriques, Francis Ricardo dos Reis Justi

**Affiliations:** Federal University of Juiz de Fora, Juiz de Fora, Brazil

**Keywords:** Phonological awareness, Phonemic awareness, Dimensionality, Confirmatory factor analysis, Reading

## Abstract

Phonological awareness is one of the most important predictors of reading. However, there is still controversy concerning its dimensionality. This study evaluated the dimensionality of phonological awareness among Brazilian Portuguese-speaking children. A total of 212 children performed six phonological awareness tasks in the last year of kindergarten. Of those children, 177 performed the same tasks when they were in the first grade. The phonological awareness measures differed in both their cognitive demand (detection, blending, segmentation, and elision) and the phonological unit involved (rhyme, syllable, and phoneme). Confirmatory factor analyzes were employed to test several models of phonological awareness dimensionality. The results indicated that the best model was an oblique model of phonological units with two correlated latent factors: phonemic awareness and supraphonemic awareness. This model presented the best fit to the data both in kindergarten and in the first grade. In addition, supraphonemic awareness in the kindergarten predicted phoneme awareness in the first grade; however, phonemic awareness in the kindergarten did not predict supraphonemic awareness in the first grade. These results are compatible with phonological awareness developing from larger phonological units (e.g., syllables) to small phonological units (e.g., phonemes) and the reciprocal relationship between phonological awareness and reading. From a theoretical point of view, these results also suggest that phonological awareness is a one-dimensional construct that can be evaluated by tests employing different phonological units (e.g., syllables, rhymes, phonemes).

## Introduction

Among the basic skills related to reading and writing, phonological awareness is one of the most important (dos Santos and Barrera, 2019; Goswami, 2018). The term *phonological awareness* or *phonological sensitivity* refers to discriminating, identifying, and manipulating phonological information, be it a phoneme, rhyme, or syllable (Richardson and Nieminen, 2017). The development of phonological awareness can be described along a continuum from sensitivity of large phonological units to awareness of small phonological units. Although not in a stage-like sequence, children generally progress from mastering word-level skills to syllable-level skills and from syllable-level skills to onset–rhyme skills, with children and adults reaching phoneme level awareness only when taught to read and write (Ziegler and Goswami, 2005). Many studies have demonstrated that performance on tasks evaluating phonological awareness predicts reading and writing development (for a meta-analysis, see Melby-Lervåg, Lyster, and Hume, 2012).

Phonological awareness has been evaluated by many different tasks that differ regarding the phonological unit involved (words, rhymes, syllables, and phonemes) and their cognitive demand (detection of similarities or differences, segmentation, blending, deletion, addition, and transposition of speech sounds) (Goswami, 2018; Richardson and Nieminen, 2017). However, there is still controversy as to whether phonological awareness is a unitary ability or not (e.g., Anthony et al., 2002, 2011; Hatcher and Hulme, 1999; Papadopoulos, Kendeou, and Spanoudis, 2012; Runge and Watkins, 2006; Schatschneider, Francis, Foorman, and Fletcher, 1999; Vloedgraven and Verhoeven, 2009; Wagner et al., 1997; Wagner, Torgesen, and Rashotte, 1994; Wolff and Gustafsson, 2015). Whether phonological awareness is one-dimensional or not is an important issue because it is directly related to the type of tasks one can use to assess phonological awareness. In the present study, we test several theoretical models on the dimensionality of phonological awareness in Brazilian Portuguese. Also, we evaluate whether the same model would describe the phonological awareness dimensionality in kindergarten and at the end of first grade. To do this, first, we review the research about phonological awareness dimensionality internationally and with Brazilian Portuguese speakers. After this, we present the rationale for the present study and its goals.

## International studies on phonological awareness dimensionality

One of the first studies about phonological awareness dimensionality was conducted by Stanovich, Cunningham, and Cramer (1984). In that study, 49 kindergarten children performed phonological awareness tasks differing in the unit of sound involved (rhyme and phoneme) and in their cognitive demand (production, detection, substitution, and deletion). The researchers performed an exploratory factor analysis considering the scores on the ten tasks administered and identified evidence of only one factor, which accounted for 48% of the variance in the data.

Wagner, Torgesen, Laughon, Simmons, and Rashotte’s (1993) study was one of the first to report evidence that phonological awareness may not be a one-dimensional construct. The study was conducted with 95 kindergartners and 89 second-graders. In the study, several phonological awareness tasks were administered, such as phoneme deletion, rhyme and alliteration detection, phoneme segmentation, blending onset and rhyme, blending phonemes into words, and blending phonemes into nonwords. Also, confirmatory factor analysis models were constructed to identify the model most consistent with the data. The results indicated that the two-correlated-factor model, with one factor indexed by three measures of blending (termed “phonological synthesis”) and another indexed by the other measures of phonological awareness (termed “phonological analysis”), demonstrated the best fit to the data.

Because of the inconsistency of the initial findings on the dimensionality of phonological awareness, several other studies on this topic were conducted, including, for example, the studies by Wagner and colleagues (Wagner et al., 1994; Wagner et al., 1997); Anthony and colleagues (Anthony et al., 2002; Anthony et al., 2011); Schatschneider et al. (1999); Hatcher and Hulme (1999); Runge and Watkins (2006); Papadopoulos and colleagues (Papadopoulos, Spanoudis, and Kendeou, 2009; Papadopoulos et al., 2012); and Vloedgraven and Verhoeven (2009). The results were mixed, with some studies corroborating the one-dimensionality of phonological awareness (Anthony et al., 2002, 2011; Papadopoulos et al., 2012; Schatschneider et al., 1999; Vloedgraven and Verhoeven, 2009; Wagner et al., 1997) and some studies corroborating the two-dimensionality of the construct (Hatcher and Hulme, 1999; Runge and Watkins, 2006; Wagner et al., 1994; Wolff and Gustafsson, 2015).

It should be noted that despite presenting consistent results, the studies that corroborated the one-dimensionality of phonological awareness (Anthony et al., 2002, 2011; Papadopoulos et al., 2012; Schatschneider et al., 1999; Vloedgraven and Verhoeven, 2009; Wagner et al., 1997) differed significantly regarding either the participants’ language, age, or the phonological awareness tasks administered. The languages included Dutch (Vloedgraven and Verhoeven, 2009), Greek (Papadopoulos et al., 2012), Spanish (Anthony et al., 2011), and English (Anthony et al., 2002; Schatschneider et al., 1999; Wagner et al., 1997). Considering age, for example, the study by Anthony et al. (2002) was conducted with younger children (2 and 3 years of age), and the study by Wagner et al. (1997) was conducted with older children who were already in the 4^th^ grade. Most phonological awareness tasks included rhyme and phoneme awareness measures, with cognitive demand varying among the studies. Furthermore, and most importantly, the one-dimensional models were different across studies. For example, in Anthony et al. (2011), the best model was characterized by four factors related to the tasks’ cognitive demand (blending multiple-choice, blending free-response, elision multiple-choice, and elision free-response) loaded on a single, second-order factor. However, in Papadopoulos et al.’s (2012) study, the phonological awareness dimensionality was best captured by a nested-factor model, which consisted of a general first-order factor, a first-order supraphonemic factor, and a first-order phonemic factor, thus, taking into account the phonological unit involved in the tasks (e.g., rhyme, syllable, and phoneme).

On the other hand, the studies that reported evidence refuting the one-dimensionality of phonological awareness (Hatcher and Hulme, 1999; Runge and Watkins, 2006; Wagner et al., 1994) were also not consistent regarding the types of factors identified. For example, Wagner et al. (1994) replicated the findings of Wagner et al. (1993): two factors were identified, one termed “phonological synthesis” (indexed by the items of the measures of blending) and another termed “phonological analysis” (indexed by the items of the measures of phoneme deletion, rhyme, and alliteration detection and phoneme segmentation). In turn, in the study by Runge and Watkins (2006), although the researchers also identified two factors, the first factor included measures of phoneme detection and manipulation, and the second factor included measures of rhyme. Thus, whereas cognitive demand (analysis versus synthesis) defined the difference between the factors in the studies by Wagner et al. (1993, 1994), in Runge and Watkins, it was the phonological unit involved in the tasks (rhyme versus phoneme).

Hatcher and Hulme (1999) presented results more consistent with Runge and Watkins because, in their study, the measure of rhyme loaded on a factor different from the measures involving phoneme deletion, blending, and segmentation. Besides, recently, Wolff and Gustafsson (2015) employed phonological awareness tasks varying in cognitive demand (identification, blending/segmentation, and manipulation) and phonological unit (morpheme/word, syllable, and phoneme) and used confirmatory factor analysis to test different models about phonological awareness dimensionality. Although they refuted a one-dimensional model, their results corroborated a bifactor model with a general factor and four narrow factors representing the cognitive demand (blending/segmentation and manipulation) and the phonological unit (morphemes/words and phonemes) involved in the tasks.

Further complicating the issue of phonological awareness dimensionality, we shall consider that the degree of correspondence between graphemes and phonemes of a writing system can vary. This variation can affect the relationship between phonological awareness and reading (e.g., Ziegler et al., 2010). According to the psycholinguistic grain size theory (Ziegler and Goswami, 2005), since phonological awareness has a reciprocal relationship with reading, consistency in grapheme-phoneme mapping affects the development of phonological awareness (allowing the refinement of phonemic awareness). Consequently, it is possible that the dimensionality of phonological awareness could also be affected by the language’s writing system. Thus, research about the dimensionality of phonological awareness in understudied languages like Brazilian Portuguese is an important source of evidence for this debate.

According to Seymour, Aro, and Erskine (2003) classification, Portuguese has a simpler syllabic structure than Swedish and Dutch and is in an intermediate position concerning the consistency in the grapheme-phoneme mapping (between English, which has the less consistent grapheme-phoneme mapping, and Greek and Spanish, which have very consistent grapheme-phoneme mappings). Therefore, considering the languages in which phonological awareness dimensionality was studied so far (Greek—Papadopoulos et al., 2012, Spanish—Anthony et al., 2011, Swedish—Wolff and Gustafsson, 2015, Dutch—Vloedgraven and Verhoeven, 2009, and English—e.g., Anthony et al., 2002 and Hatcher and Hulme, 1999), Brazilian Portuguese adds information on variations in syllabic structure and consistency in grapheme-phoneme mapping.

### Studies with Brazilian Portuguese

Regarding studies conducted with Brazilian Portuguese speakers, to our knowledge, three studies investigated the factorial structure of different measures of phonological awareness: Godoy and Cogo-Moreira (2015); Santos and Lima (2017); and Germano, César, and Capellini (2017). The study by Godoy and Cogo-Moreira (2015) included three measures of phonemic awareness (phoneme segmentation, onset deletion in nonwords with a consonant-vowel-consonant [CVC] structure, and onset deletion in nonwords with a consonant-consonant-vowel [CCV] structure); this study was conducted with 176 Brazilian 1^st^ to 5^th^ graders. The researchers assessed the fit of the data to a model in which the items of each of the three phonological awareness measures represented different factors that were correlated (segmentation, CVC deletion, and CCV deletion). The results indicated a good fit of this model to the data. The correlation among the three factors was strong. However, the authors did not compare the three-factor model with a one-factor model to assess which model best fits the data.

Santos and Lima (2017) aimed to investigate the evidence of construct validity of a phonological awareness instrument, the *Roteiro de Avaliação da Consciência Fonológica* (RACF). The RACF comprises three sections of items, each with five items to evaluate difficulties in detecting a phoneme at the beginning, middle, and end of words. Although the authors expected a single factor in the instrument, exploratory and confirmatory factor analysis suggested three factors in the RACF. These results are difficult to interpret theoretically because there is not a manipulation of phonological unit (e.g., syllable X phoneme) or cognitive demand (e.g., analysis X synthesis) in the RACF. Thus, as Santos and Lima acknowledge, the factors could be a byproduct of item difficulty (e.g., differential difficulty in detecting a phoneme at the beginning, end, and middle of the words).

Germano et al. (2017) evaluated the dimensionality of phonological awareness in the context of a screening protocol to identify children at risk of dyslexia. The study included several phonological awareness measures comprising rhyme production, rhyme identification, syllabic segmentation, production of words from a given phoneme, phonemic synthesis, and phonemic analysis. A total of 149 children aged 6 years to 6 years and 11 months were evaluated in phonological awareness and several other measures. An exploratory factor analysis was carried out, and four factors were retained. More importantly, the phonological awareness measures loaded mainly in two factors, one with measures of rhyme, alliteration and letter-naming knowledge, and the other with measures of phoneme analysis and synthesis, together with rapid automatized naming and word and nonword decoding. These results suggest that the phonological unit involved in the tasks (e.g., phonemic X supraphonemic) could be responsible for the two-dimensionality of phonological awareness. However, it is important to notice that syllabic segmentation did not load in the same factor as rhyme and alliteration. Besides, the inclusion of other variables not theoretically related to phonological awareness in the analysis makes it difficult to interpret the factors.

### The present study

Considering the divergence among the results from studies on the dimensionality of phonological awareness conducted thus far in Brazil (Germano et al., 2017; Godoy and Cogo-Moreira, 2015; Santos and Lima, 2017), and internationally (Anthony et al., 2002, 2011; Hatcher and Hulme, 1999; Papadopoulos et al., 2012; Runge and Watkins, 2006; Schatschneider et al., 1999; Vloedgraven and Verhoeven, 2009; Wagner et al., 1997; Wagner et al., 1994; Wolff and Gustafsson, 2015), we aim to investigate this issue. The present study included measures that differ in their cognitive demand (detection, blending, segmentation, and deletion) and the phonological unit (rhyme, syllable, and phoneme). Thus it allows assessing whether measures that differ only in their cognitive demand (e.g., blending compared with segmentation) or in the linguistic unit emphasized (e.g., syllable compared with phoneme) will be in the same factor or different factors. To investigate this issue, confirmatory factor analysis was employed to test five models. One model representing a strong one-dimensional hypothesis: a one-dimensional model with all measures indexing a single factor. Two oblique models representing weak one-dimensional hypotheses: a two-dimensional model with two correlated factors representing phonological units (phonemic X supraphonemic); and a two-dimensional model with two correlated factors representing cognitive demand (analysis X synthesis). Two orthogonal models tested strong two-dimensional hypotheses: a two-dimensional model with two uncorrelated factors representing phonological units (phonemic X supraphonemic); and a two-dimensional model with two uncorrelated factors representing cognitive demand (analysis X synthesis).

In addition, the present study also investigates longitudinally whether phonological awareness dimensionality would be the same in kindergarten and at the end of the first grade (formal reading instruction starts in Brazil in the first grade). Thus, it allows evaluating the effect of reading instruction on phonological awareness dimensionality. It is important to do this because, according to the psycholinguistic grain size theory (Ziegler and Goswami, 2005), phonological awareness has a reciprocal relationship with reading and phonemic awareness only develops along with the teaching of reading.

We have no knowledge of other studies that have conducted a comprehensive investigation of phonological awareness dimensionality in Brazilian Portuguese like this one. Theoretically, it is important to have data on the dimensionality of phonological awareness in different languages because this can clarify whether phonological awareness is a general metalinguistic ability that underlies the acquisition of literacy in any alphabetical language (Khalaf, Santi, Kulesz, Bunta, and Francis, 2019). From a more practical point of view, it is essential to evaluate the dimensionality of phonological awareness because it has implications for test building and psychological assessment in terms of the kind of items employed in phonological awareness’ tasks and their interpretation.

## Method

### Participants

This study included 212 Brazilian children enrolled in the last year of kindergarten. Of the 212 children, 99 were enrolled in private schools, including 47 boys and 52 girls; and 113 were enrolled in public schools, including 55 boys and 58 girls. Considering the entire sample, the children’s mean age at the beginning of the study was approximately 6 years (72.2 months), with a standard deviation of 3.7 months. All children had Brazilian Portuguese as their native language and were from nine private schools and seven public schools from different regions in a city of approximately 500,000 inhabitants in southeastern Brazil. Of the 212 children who participated in the study at time 1 (last year of kindergarten), 177 performed the same phonological awareness tasks at time 2 when they were in the first grade. The main reasons for the loss of participants from time 1 to time 2 were children who changed schools; children who missed one of the testing sessions; and children who did not want to participate in time 2. The children’s mean age at time 2 was approximately 6 years and 8 months (80.2 months), with a standard deviation of 3.6 months. There were 89 boys and 88 girls in time 2.

The inclusion criterion was the signing of the Informed Consent Form by the children's guardians. The exclusion criterion was a parental report of diagnosis of intellectual disability, including, for example, Down syndrome and fragile X syndrome, or the presence of uncorrected sensory impairments such as blindness or deafness. The exclusion criterion for time 2 was the same for time 1. None of the children needed to be excluded from time 2 due to these exclusion criteria. Thus we are assuming a typical developing sample in the present study.

### Procedures and materials

Each phonological awareness task comprised three training items and 10 test items. For all tasks, in the three training items, feedback regarding the correctness of participant answers was included to ensure that all participants knew what was expected of them. The words for all tasks were sampled from Pinheiro’s word count (Pinheiro, 1996) to avoid unusual items within each task. Each item answered correctly scored one point on all tasks. The selection and development of these tasks considered the level of difficulty and the linguistic complexity used in previous studies which assessed phonological awareness dimensionality (e.g., Anthony, Lonigan, Driscoll, Phillips, and Burgess, 2003; Anthony and Lonigan, 2004; Papadopoulos et al., 2012; Wolff and Gustafsson, 2015) or in Brazilian Portuguese studies (e.g., Cardoso-Martins, 1995; Germano et al., 2017; Roazzi, Roazzi, Justi, and Justi, 2013). The tasks were developed by the authors’ research group.

#### Rhyme detection task

This task requires the child to say which of three words presented orally and, concomitantly, in images have a similar final sound, that is, that rhyme. For example, after being presented with the words /pwte/[POT], /bwte/ [BOAT] and /vaka/ [COW], the child should say /pwte/ and /bwte/. The items were arranged at a level of increasing difficulty, considering the number of shared phonemes between the distractor and targets. Based on the present study’s data, this task had a Cronbach’s alpha reliability coefficient of .55.

#### Syllable blending task

In this task, each target word is pronounced, including a 1-s pause between each syllable (/Ra/; /to/), and the child is asked to join the syllables mentally and to say the resulting word (/Rato/, [RAT]). Based on the present study’s data, this task had a Cronbach’s alpha reliability coefficient of .92.

#### Syllable segmentation task

This task requires the child to segment the words spoken by the experimenter (for example: /Rato/, [RAT]) into their respective syllables (/Ra/; /to/), using images to help the child in this task. Based on the present study’s data, this task had a Cronbach’s alpha reliability coefficient of .88.

#### Phoneme blending task

In this task, isolated phonemes are presented (/R/; /a/; /t/; /o/), and the child is asked to mentally join them and say the resulting word (/Rato/, [RAT]). Based on the present study’s data, this task had a Cronbach’s alpha reliability coefficient of .88.

#### Phoneme segmentation task

This task requires the child to say the phonemes heard (for example: /R/; /a/; /t/; /o/) in the words spoken by the experimenter (for example: /Rato/, [RAT]). Figures are used in this task to help the child visually. Based on the present study’s data, this task had a Cronbach’s alpha reliability coefficient of .88.

#### Phoneme elision task

This task consists of orally presenting a word (for example: /Rato/, [RAT]) and requires the participant to mentally delete a specific sound pronounced by the experimenter (for example: /R/) and say the remaining sound (for example: /ato/). Based on the present study’s data, this task had a Cronbach’s alpha reliability coefficient of .87.

After the schools were contacted and permission to conduct the study in their facilities was granted, all children from the final year of kindergarten who wanted to participate in the study and whose parents authorized their participation performed the phonological awareness tasks described above. The tasks were administered in the last three months of the school year, on days and at times agreed upon with the school administrators and teachers. Each child participated in one individual session of approximately 30 min each.

### Ethics statement

This study is part of a broader longitudinal study approved by the Human Research Ethics Committee of the authors’ institution (Protocol: 16525513.9.0000.5147). Procedures in this study adhered to ethical research policies and were also approved by the children’s schools board. In addition to the signing of the Informed Consent Form by the children’s guardians, oral assent was obtained from each child at every testing session.

## Results

Data on the maximum possible score of the task, the maximum score obtained, the minimum score obtained, mean, standard deviation, skew, and kurtosis obtained in the phonological awareness tasks are outlined in Table 1.

**Table 1:** Descriptive statistics of the phonological awareness tasks

	N	Chance	Mx. T.	Mx. O.	Mn. O.	M (SD)	Skew	Kurtosis
RhyDet_T1	212	3.33	10	10	0	4.62 (2.11)	− 0.22	− 0.01
SyllBlen_T1	212	0	10	10	0	7.72 (3.16)	− 1.41	0.75
SyllSeg_T1	212	0	10	10	0	6.74 (3.16)	− 0.76	− 0.58
PhonBlen_T1	212	0	10	10	0	0.56 (1.60)	3.39	12.05
PhonSeg_T1	212	0	10	8	0	0.26 (1.09)	4.92	25.20
PhonEli_T1	212	0	10	9	0	0.40 (1.33)	4.27	19.28
RhyDet_T2	177	3.33	10	10	0	5.44 (2.29)	− 0.32	0.15
SyllBlen_T2	177	0	10	10	0	7.84 (3.50)	− 1.53	0.70
SyllSeg_T2	177	0	10	10	0	8.15 (2.63)	− 1.70	1.99
PhonBlen_T2	177	0	10	9	0	1.53 (2.51)	1.61	1.44
PhonSeg_T2	177	0	10	10	0	0.70 (1.94)	3.07	9.36
PhonEli_T2	177	0	10	10	0	1.05 (2.28)	2.33	4.75

*RhyDet* Rhyme detection, *SyllBlen* Syllable blending, *SyllSeg* Syllable segmentation, *PhonBlen* Phoneme blending, *PhonSeg* Phoneme segmentation, *PhonEli* Phoneme Elision, *T1* kindergarten, *T2* first grade, *N* number of children who performed the task, *Chance* possible score by chance, *Mx*. *T*. maximum score of the task, *Mx*. *O*. maximum score obtained, *Mn*. *O*. minimum score obtained, *M* mean, *SD* standard deviationan

As presented in Table 1, the scores of the measures rhyme detection, syllable blending, and syllable segmentation had higher variability than the scores on phonemic awareness measures. The Children performed above chance level in all tasks in the kindergarten (T1) and first grade (T2) (one-sample *T* tests, all *p* values < 0.01). Except for syllable blending, as expected, all scores on phonological awareness tasks increased from kindergarten (T1) to first grade (T2) (Wilcoxon signed ranks tests, all *p* values < 0.001).

To identify a possible sample bias because of the loss of participants between T1 and T2, the means of children who participated in T1 and in T2 and the means of children who participated only in T1, in the T1 phonological awareness tasks, were compared. This comparison was conducted using the Mann-Whitney *U* test. The results indicated no significant difference in performance in the rhyme detection (*z* = − .801, *p* = .42), syllable blending (*z* = − .198, *p* = .84), syllable segmentation (*z* = − 0.284, *p* = .777), phoneme blending (*z* = − .295, *p* = .77), phoneme segmentation (*z* = − .544, *p* = .58), and phoneme elision (*z* = − 1.00, *p* = .31) tasks between these two groups of children.

### The dimensionality of phonological awareness

First, it was assessed the internal consistency of the items in each phonological awareness task. Except for rhyme detection, which had a poor Cronbach’s alpha reliability coefficient (.55), all the other measures presented very good Cronbach’s alpha reliability coefficients: syllable blending (.92), syllable segmentation (.88), phoneme blending (.88), phoneme segmentation (.88), and phoneme elision (.87). Thus, the score on the rhyme detection task was excluded from all the following analysis.

Five confirmatory factor analysis models were constructed to assess whether measures that differ in their cognitive demand (blending compared with segmentation) or in the linguistic unit emphasized (syllable compared with phoneme) would be best represented by a one-dimensional or by a two-dimensional model. Two oblique models representing weak one-dimensional hypotheses were constructed: Model 2FO:AxS and Model 2FO:PxSP. Model 2FO:AxS reflects the cognitive demands of the tasks and is represented by two intercorrelated latent factors: phonological analysis (syllable segmentation, phoneme segmentation, and phoneme elision) and phonological synthesis (syllable blending and phoneme blending). Model 2FO:PxSP,reflects the phonological units of the tasks and is represented by two intercorrelated latent factors: phonemic units (phoneme blending, phoneme segmentation, and phoneme elision) and supraphonemic units (syllable segmentation and syllable blending). In addition, to test a strong one-dimensional hypothesis, Model 1F was constructed in which a single factor captured syllable blending, syllable segmentation, phoneme blending, phoneme segmentation, and phoneme elision tasks. Model 1F is nested under the two-factor models because it can be obtained by restricting the two latent factors' intercorrelations to 1. Model 1F is also the most parsimonious one and shall be preferred if it explains the data equally well compared with the two-factor models.

To test whether phonological awareness would be best represented by a two-dimensional model, two orthogonal models tested strong two-dimensional hypotheses. These two models were constructed based on the models 2FO:AxS and 2FO:PxSP, by restricting the intercorrelations between the two latent factors to 0: 2FOrt:AxS and 2FOrt:PxSP, respectively. Model 2FOrt:AxS reflects the cognitive demands of the tasks and is represented by two uncorrelated latent factors: phonological analysis (syllable segmentation, phoneme segmentation, and phoneme elision) and phonological synthesis (syllable blending and phoneme blending). Model 2FOrt:PxSP reflects the phonological units of the tasks and is represented by two uncorrelated latent factors: phonemic units (phoneme blending, phoneme segmentation, and phoneme elision) and supraphonemic units (syllable segmentation and syllable blending).

As shown in Table 1, especially in time 1, departures from normality were found for a reasonable number of variables. Thus to evaluate the models, maximum likelihood robust methods were employed, and Bollen-Stine bootstrap with 2000 bootstrap samples (Bollen and Stine, 1992) was used to correct the chi-square (*X*^2^). Following Cangur and Ercan’s (2015) criteria of model fit, a good fit to the data was indicated by (a) a nonsignificant chi-square test (in the present study, we employed the Bollen-Stine bootstrapped *X*^*2*^); (b) a comparative fit index (CFI) with values of at least .95; and (c) a root mean square error of approximation (RMSEA) with values of .08 or less. In addition, to compare different nested models, we tested the difference in their Bollen-Stine bootstrapped *X*^2^ (a nonsignificant test indicates that the restrictive model fits the data just as well as the less restrictive comparison model), and we report two information criteria indices, namely the Akaike information criterion (AIC) and the Bayesian information criterion (BIC), where smaller values represent a better fitting model. Table 2 presents the fit indices for the five different models when the children were in the kindergarten.

**Table 2:** Fit indices for models of the structure of phonological awareness tasks

Model	B-S *X*^*2*^	*df*	CFI	RMSEA	B-S *X*^*2*^ difference with Model 1F	AIC	BIC
1F	6.916	5	.998	.020	–	25.443	59.009
2FO:AxS	6.593	4	.995	.041	.322, *p* = 0.570	27.392	64.314
2FO:PxSP	1.188	4	> .999	< .001	5.727, *p* = 0.017	22.749	23.393
2FOrt:AxS	22.458*	6^a^	.445	.349	–	177.946	208.155
2FOrt:PxSP	13.002*	6^a^	.975	.074	–	31.013	61.222

*B*-*S X*^2^ Bollen-Stine bootstrapped *X*^*2*^, *df* degrees of freedom, *CFI* Comparative Fit Index, *RMSEA* root mean square error of approximation, *AIC* Akaike information criterion, *BIC* Bayesian information criterion

*p < .05

^a^It was necessary to impose an additional restriction on the model, so that it could be identified

As can be seen in Table 2, considering the data from children in the last year of kindergarten (*N* = 212), the strong two-dimensional orthogonal models (models 2FOrt:AxS and 2FOrt:PxSP) presented a statistically significant B-S *X*^2^ (all *p* values < .05), suggesting a poor fit to the data. Concerning the one-dimensional models (strong and weak), all three models presented a good fit to the data with a nonsignificant B-S *X*^2^ (all *p* values > .15), CFI values higher than .95, and RMSEA values lower than .08 (Cangur and Ercan, 2015). These results suggest Model 1F as the best choice due to its parsimony, especially in comparison with Model 2FO:AxS, which did not present a statistically significant smaller B-S *X*^2^ value and presented higher AIC and BIC values. However, the situation is different when Model 1F is compared to Model 2FO:PxSP, because the latter presented a B-S *X*^2^ significantly lower than model 1F (B-S *X*^2^ difference with Model 1F = 5.727, *p* = 0.017), indicating a better data fit for Model 2F:PxSP. It is important to notice that smaller AIC and BIC values represent a better fitting model, and Model 2F:PxSP also presented smaller AIC and BIC values than Model 1F. Thus, at least in the last year of kindergarten, the model that best represents phonological awareness dimensionality in Brazilian Portuguese is a weak one-dimensional model in which phonological units represent two intercorrelated latent factors: phonemic and supraphonemic.

**Fig. 1:**
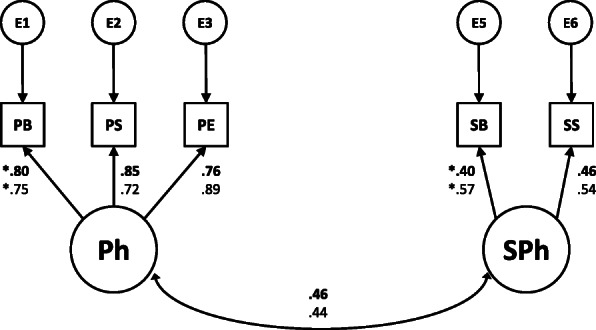
Two-factor confirmatory model of phonological awareness (Model 2F:PxSP). Parameters represent standardized estimates for children in the kindergarten (upper bold parameters) and for children in the first grade (down parameters). Factors’ correlation and all loadings from the latent constructs (Ph, SPh) to their corresponding indicators were significant (all *p* < .05). *Ph* phonemic units, *PB* phoneme blending, *PS* phoneme segmentation, *PE* phoneme elision, *SPh* supraphonemic units, *SS* syllable segmentation, *SB* syllable blending, * metric

Taking into account the children’s data when they were in the first grade (*N* = 177), the only model that fitted the data well was the Model 2FO:PxSP (B-S *X*^2^ = 2.465, *df* = 4, *p* = .651; CFI > .999; RMSEA < .001; AIC = 23.275; BIC = 58.213). All the other models presented statistically significant B-S *X*^2^ values (all Bollen-Stine bootstrapped *p* values < .01). Thus, in the first grade, the model that best represents phonological awareness dimensionality is also the weak one-dimensional model in which phonological units represent two intercorrelated latent factors: phonemic and supraphonemic units (Model 2FO:PxSP). Figure 1 presents the model 2FO:PxSP. It is important to notice that both in the kindergarten and first grade models, the factors correlation and all loadings to their corresponding indicators were statistically significant.

In all the analyses so far, the rhyme detection task was not included because it had a poor Cronbach’s alpha reliability coefficient (.550). However, we decided to test if the model 2FO:PxSP would still present a good data fit with the inclusion of the rhyme detection task. Thus the supraphonemic factor included rhyme detection, syllable blending, and syllable segmentation. Even with the inclusion of the rhyme task, the model 2FO:PxSP fitted the data very well in the kindergarten (B-S *X*^2^ = 9.860, *df* = 8, *p* = .275; CFI = .998; RMSEA = .019; AIC = 34.590; BIC = 78.226) and in the first grade (B-S *X*^2^ = 11.960, *df* = 8, *p* = .153; CFI = .997; RMSEA = .027; AIC = 35.035; BIC = 76.325). On the other hand, with the inclusion of the rhyme task, all the other models presented statistically significant B-S *X*^2^ values (all Bollen-Stine bootstrapped *p* values < .05). Thus, the inclusion of the rhyme task does not change the conclusion that the Model 2FO:PxSP is the best model of phonological awareness dimensionality in Brazilian Portuguese.

Considering that model 2FO:PxSP was the best model in the kindergarten and the first grade, we decided to test the longitudinal relationship between the phonemic units (phoneme blending, phoneme segmentation, and phoneme elision) and supraphonemic units (syllable segmentation, and syllable blending). Paths were then added in Model 2FO:PxSP from time 1 (kindergarten) supraphonemic units to time 2 (first grade) phonemic and supraphonemic units, and paths from time 1 phonemic units to time 2 phonemic and supraphonemic units. It is important to notice that this cross-lagged longitudinal model tests two different hypotheses: (1) if there is a 3^rd^ factor mediating the relationship between the supraphonemic and phonemic factors (e.g., brain maturation or I.Q.), both time 1 supraphonemic and phonemic units shall predict time 2 supraphonemic and phonemic units; however, (2) if phonological awareness has a reciprocal relationship with reading instruction, and phonemic awareness only develops along with the teaching of reading (Ziegler and Goswami, 2005), time 1 supraphonemic units shall predict time 2 supraphonemic and phonemic units, but time 1 phonemic units shall predict only time 2 phonemic units. That is, the development of phonemic awareness depends on phonological awareness and on the teaching of reading, but supraphonemic awareness can be detected before the teaching of reading.

The longitudinal model 2FO:PxSP fitted the data well: B-S *X*^2^ = 39.078, *df* = 30, *p* = .124; CFI = .957; RMSEA = .078; AIC = 112.214; BIC = 191.618. All loadings from the latent constructs (time 1 and time 2 phonological units and supraphonological units) to their corresponding indicators were significant (all *p* < .05). More importantly, the paths from T1 supraphonemic units to T2 supraphonemic and phonemic units were significant (*p* = .002 and *p* = .06, respectively). However, considering the paths from T1 phonemic units to T2 phonemic and supraphonemic units, the former was significant (*p* < .01) but not the latter (*p* = .38). As theoretically expected, this result indicates that supraphonemic awareness is predictive of future phonemic awareness, but not otherwise.

## Discussion

The present study sought to evaluate the dimensionality of phonological awareness in Brazilian Portuguese speaking children and to assess the effects of reading instruction on phonological awareness dimensionality by investigating longitudinally whether the same model would describe phonological awareness dimensionality in kindergarten and at the end of the first grade (formal reading instruction starts in Brazil in the first grade). The results of the confirmatory factor analyses were straightforward: strong one-dimensional and two-dimensional hypotheses were refuted. The best model in kindergarten and the first grade was model 2FO:PxSP, a weak one-dimensional model with two intercorrelated latent factors representing supraphonemic and phonemic units.

Overall, we believe that the results are in line with the Psycholinguistic Grain Size Theory (Ziegler and Goswami, 2005). For example, the model 2FO:PxSP supraphonemic and phonemic latent factors are compatible with the idea that phonological awareness development is quasi-hierarchical (e.g., Anthony et al., 2003; Goswami, 2018), that is, awareness of larger grain sizes (e.g., words, syllables, rhymes) develops first, and phonemic awareness only develops once children are taught to read and write (Ziegler and Goswami, 2005). This developmental pattern is evident in the children's scores: although all scores were above chance level, children performed very well on syllabic tasks but performed very poor on phonemic tasks.

Performance at floor level on the phoneme tasks employed in the present research is a common finding for six-year-old children (e.g., Anthony et al., 2003; Papadopoulos et al., 2012). It is interesting to note that, although children increased their scores in the phoneme tasks from kindergarten to first grade, about eight months later, their scores were still very low. An interesting contrast is to compare these results with those from the study of Papadopoulos et al. (2012) with Greek children because they also employed a phoneme elision task, and participants from their study are about the same age as ours in time 1 and in time 2. In both studies, it is interesting to note that, in time 1, children were at floor levels on the phoneme elision task. However, 1 year later, children from Papadopoulos et al.’s study were at the mean score in the task while children from our study were still struggling. We believe that this difference can be explained, at least in part, by differences between the languages in grapheme-phoneme mapping. After all, Greek is much more consistent than Portuguese, and learning to read is expected to develop faster in consistent languages (e.g., Seymour et al., 2003; Ziegler et al., 2010). According to Psycholinguistic Grain Size Theory, this consistency effect occurs because phonological representations become amalgamated with orthographic representations as literacy is acquired (Ziegler and Goswami, 2005). Thus, it is easier for children to discover and isolate phonemes as they learn to read in consistent orthographies.

The present study also tested the longitudinal relationship between the 2FO:PxSP model’s phonemic and supraphonemic awareness latent factors. The supraphonemic awareness in kindergarten predicted phonemic awareness in the first grade; however, phonemic awareness in kindergarten did not predict supraphonemic awareness in the first grade. Since a cross-lagged model was employed, we could reject a common 3^rd^ factor explanation (if that was the case, we could expect both time 1 factors to predict both time 2 factors to some degree). We interpret this finding in line with the reciprocal relationship between reading and phonological awareness in the sense that a certain level of phonological awareness may be necessary for grasping the alphabetic principle per se, and, as the learning of grapheme-phoneme mappings progress, this knowledge promotes the refinement of phonemic awareness (Ziegler and Goswami, 2005). Thus, it makes sense to think that supraphonemic awareness could predict initial phoneme awareness. However, as phonemic awareness is also influenced by learning to read, phonemic awareness increases due to reading experience are not expected to predict future supraphonemic awareness.

Considering other studies that investigated the factorial structure of different phonological awareness measures with Brazilian Portuguese speaking children (Godoy and Cogo-Moreira, 2015; Santos and Lima, 2017; Germano et al., 2017), the most comparable to ours is the study by Germano et al. (2017). The study by Godoy and Cogo-Moreira (2015) and the study by Santos and Lima (2017) did not manipulate the linguistic unit involved in the phonological awareness tasks in their study (they only employed phonemic tasks) and investigated older children (from first to 6^th^ grade). Especially in Santos and Lima’s study, the results are difficult to interpret because there is no manipulation of phonological unit (e.g., syllable X phoneme) or cognitive demand (e.g., analysis X synthesis). The study by Godoy and Cogo-Moreira somehow manipulated the tasks’ cognitive demand (segmentation X deletion tasks). However, it is important to notice that their three factors were strongly correlated (*r* = 0.74 to *r* = 0.83), suggesting that a unidimensional model would be consistent with the data (unfortunately, the researchers did not assess the data fit of a one-dimensional model).

On the other hand, the results by Germano et al. (2017) study are consistent with ours, because the phonological awareness measures loaded mainly in two factors, one with supraphonemic measures (e.g., rhyme and alliteration) and one with measures of phoneme analysis and synthesis. We attribute the fact that syllabic segmentation in Germano et al.’s study did not load in the same factor as rhyme and alliteration to the inclusion of other measures in the analyses not direct related to the construct of phonological awareness (e.g., letter-name knowledge, rapid automatized naming, word and nonword reading, etc.). Another problem was the use of exploratory factor analysis which do not allow the test and comparison of specific hypotheses about phonological awareness dimensionality. Thus, we believe that the present research advances in relation to previous research in Brazilian Portuguese by testing different theoretical models about phonological awareness dimensionality and by demonstrating that a weak one-dimensional model with correlated latent factors representing supraphonemic and phonemic awareness (model 2FO:PxSP) is the best model both in kindergarten and in first grade.

Regarding international studies, the results of the present study are consistent with the one-dimensional interpretations of the results from the studies by Anthony et al. (2002, 2011), Anthony and Lonigan (2004), Lonigan et al. (Lonigan, Burgess, Anthony, and Barker, 1998; Lonigan, Burgess, and Anthony, 2000), Papadopoulos et al. (Papadopoulos et al., 2009; Papadopoulos et al., 2012), Schatschneider et al. (1999), Vloedgraven and Verhoeven (2007, 2009), and Wagner et al. (1997). Consider, for example, Anthony and Lonigan (2004) study, which reanalyzed data from four different studies with English speaking children. Anthony and Lonigan employed confirmatory factor analyses and reported that the difference between fits of the nested one-factor and two-factor oblique models was not significant in the kindergarten and the first grade. However, the two-factor oblique model fit was reliably better than that of the one-factor model in the second-grade data. It is interesting to notice that the conceptualization of the two-factor oblique model from Anthony and Lonigan’s study is compatible with two correlated supraphonemic and phonemic factors like the model 2FO:PxSP in the present study (the main difference being that, in Anthony and Lonigan’s study, the supraphonemic factor was indexed by onset and rhyme variables).

Another interesting example comes from the study by Papadopoulos et al. (2012) with Greek speaking children. The findings from Papadopoulos et al. about phonological awareness dimensionality favored a nested-factor model, which consisted of a general first-order factor, a first-order supraphonemic factor, and a first-order phonemic factor, representing a single unified construct. Thus, the conceptualization of phonological awareness consisting somehow of supraphonemic and phonemic factors seems to have empirical support in languages that differ significantly in grapheme-phoneme mapping: Greek (Papadopoulos et al., 2012) being the most consistent, Portuguese a kind of middle ground, and English (Anthony and Lonigan, 2004) being by far the least consistent language. However, it is important to point out that not all studies that favored unidimensional interpretations of phonological awareness considered the tasks’ phonological unit as one of its facets. For example, in the study by Anthony et al. (2011), the phonological awareness facets were characterized by the tasks’ cognitive demand (blending multiple-choice, blending free-response, elision multiple-choice, and elision free-response).

On the other hand, the results of the present study are inconsistent with the findings of Wagner et al. (1993, 1994), Hatcher and Hulme (1999), Runge and Watkins (2006), and Wolff and Gustafsson (2015). A reason for the difference in results regarding the studies by Wagner and colleagues may be that those researchers did not assess a second-order model. The studies by Wagner et al. (1997) and Wagner et al. (1994) evaluated the same sample. In the 1994 study, data on children from kindergarten to 2^nd^ grade were analyzed. In the 1997 study, data on the 2^nd^ graders were reanalyzed, together with data on those same children when they were in the 3^rd^ and 4^th^ grades. A strong correlation was identified between the factors “phonological synthesis” and “phonological analysis”; the correlations were equal to or stronger than 0.78 in the initial years and reached perfection with data on the 3^rd^ graders. Thus, in the 1997 study, those researchers decided to assess the fit to the data of a model with a second-order factor and determined that it was consistent with the data in all analyses (kindergarten, 1^st^, 2^nd^, 3^rd^, and 4^th^). Therefore, because the same children were analyzed in the studies by Wagner et al. (1994) and Wagner et al. (1997), the two-dimensionality identified in the previous studies by Wagner and colleagues may be because alternative models representing one-dimensionality were not tested.

The inconsistency between the results of the present study and the findings of Hatcher and Hulme (1999) and Runge and Watkins (2006) can be attributed to differences in the statistical analysis employed because, in both studies, phonological awareness was conceptualized as involving supraphonemic (mainly rhyme) and phonemic factors. For example, both Hatcher and Hulme and Runge and Watkins employed exploratory factor analysis and included measures of other constructs in the analyses. The measures of other constructs and the kind of rotation employed can obscure factor interpretation in studies with exploratory factor analysis (Anthony and Lonigan, 2004). Also, it is important to point out that in the study by Runge and Watkins the correlation between the rhyme factor and the phonemic awareness factor was .63; thus, it does not exclude a one-dimensional interpretation. Therefore, the difference in results between the present study and the studies by Hatcher and Hulme and by Runge and Watkins can be attributed to the type of statistical analysis employed (e.g., exploratory factor analysis versus confirmatory factor analysis).

Wolff and Gustafsson (2015) is another interesting study to compare with ours. Wolff and Gustafsson’s findings corroborated a bifactor model with a general factor and four narrow factors representing the cognitive demand (blending/segmentation and manipulation) and the phonological unit (morphemes/words and phonemes) involved in the tasks. Thus, to some extent, their results diverge from ours because they had to include a factor accounting for the tasks’ cognitive demand. However, Wolff and Gustafsson also tested a structural equation model of how the cognitive abilities Visual (e.g., Colored Progressive Matrices, Wechsler Matrices), Verbal (e.g., Word Span forward and backward), and Fluid Intelligence relate to the phonological awareness factors representing cognitive demand and phonological unit. In this model, when the relationship between fluid intelligence and phonological awareness was considered, factors related to the tasks’ cognitive demand (identification, blending/segmentation, and manipulation) were significantly related to fluid intelligence, but the factors related to the tasks’ phonological units (syllables and phonemes) were not. In addition, the phonological units (phonemes and syllables) had a moderate correlation with verbal ability. If we consider that the measures of verbal ability involved mainly measures related to phonological working memory (e.g., word span forward and backward), this result can be interpreted as convergent validity evidence for phonological units as an important facet of phonological awareness because phonological awareness and phonological working memory are expected to correlate in a phonological processing model (e.g., Wagner et al., 1993).

On the other hand, since phonological awareness and fluid intelligence are expected to be different constructs, the lack of relationship between fluid intelligence and factors related to the tasks’ phonological units in Wolff and Gustafsson (2015)’ study can be interpreted as discriminant validity evidence for these factors. Therefore, we interpret Wolff and Gustafsson results as indicating that the type of phonological unit is a true facet of phonological awareness, while cognitive demand may be only accidentally related to it. In this regard, Wolff and Gustafsson results are quite compatible with ours.

### Present study’s limitations

A limitation of the present study was the rhyme detection task which had a poor Cronbach’s alpha reliability coefficient (.550) and was not included in the confirmatory factor analyses. It is not clear why the rhyme task presented a low reliability coefficient, although we suspect that some items were very difficult because they shared many phonemes with the distractor word. However, it is important to notice that the inclusion of the rhyme detection task did not change the results, that is, Model 2FO:PxSP was again the model which presented the best fit to the data. Thus, the inclusion of the rhyme task does not change the conclusion that the model that best represents phonological awareness dimensionality in Brazilian Portuguese is the weak one-dimensional model in which phonological units represent two intercorrelated latent factors: phonemic units and supraphonemic units (Model 2FO:PxSP).

Another limitation of the present study is that we have assumed a typical developing sample based on the lack of parental report of diagnosis of intellectual disability. However, a much stronger conclusion concerning these matters would be achieved if we had employed an intelligence test in the present research. Thus, it would be interesting if future studies investigate the dimensionality of phonological awareness in typical and atypical developing children. As far as we know, there is no such study.

## Conclusion

Phonological awareness is one of the most important predictors of reading. However, there is still controversy as to whether phonological awareness is a unitary ability or not. In this study, we employed phonological awareness measures that differ both in cognitive demand (detection, blending, segmentation, and elision) and in the phonological unit involved (rhyme, syllable, and phoneme). Thus, we were able to test several theoretical models on the dimensionality of phonological awareness in Brazilian Portuguese. We also evaluated whether the same theoretical model would describe the phonological awareness dimensionality in kindergarten and in the first grade. Our results suggest that the best model of the phonological awareness dimensionality consists of two correlated factors: phonemic awareness and supraphonemic awareness. This model was the best model in kindergarten and the first grade. In addition, supraphonemic awareness in the kindergarten predicted phoneme awareness in the first grade. However, phonemic awareness in the kindergarten did not predict supraphonemic awareness in the first grade.

From a theoretical point of view, the present study’s results bring important evidence about the dimensionality of phonological awareness in Brazilian Portuguese, suggesting that, like in other languages, the one-dimensionality of phonological awareness can be assumed in Brazilian Portuguese. Our results also corroborate theories advocating that phonological awareness develops from larger phonological units to smaller phonological units. From a more practical point of view, the one-dimensionality of phonological awareness has implications for test building and psychological assessment, suggesting that tests employing different phonological units (e.g., syllables, rhymes, phonemes) can be interpreted as tapping the same underlying ability.

## Data Availability

Please contact the author for data requests.
